# Characterizing the Relationship Between the COVID-19 Pandemic and U.S. Classical Musicians' Wellbeing

**DOI:** 10.3389/fsoc.2022.848098

**Published:** 2022-03-24

**Authors:** Grace Wang, Noah R. Fram, Laura L. Carstensen, Jonathan Berger

**Affiliations:** ^1^Department of Biology, Stanford University, Stanford, CA, United States; ^2^Center for Computer Research in Music and Acoustics, Stanford University, Stanford, CA, United States; ^3^Department of Psychology, Stanford University, Stanford, CA, United States; ^4^Stanford Center on Longevity, Stanford University, Stanford, CA, United States

**Keywords:** COVID-19, mental wellbeing, social isolation, SARS-CoV-2, musicians

## Abstract

The COVID-19 pandemic has devastated the economic and social wellbeing of communities worldwide. Certain groups have been disproportionately impacted by the strain of the pandemic, such as classical musicians. The COVID-19 pandemic has greatly harmed the classical music industry, silencing the world's concert halls and theaters. In an industry characterized by instability, a shock as great as COVID-19 may bring negative effects that far outlast the pandemic itself. This study investigates the wellbeing of classical musicians during the COVID-19 pandemic. 68 professional classical musicians completed a questionnaire composed of validated measures of future time horizons, emotional experience, social relationships, and life satisfaction. Findings show that feelings of loneliness had a significant negative association with other measures of wellbeing and were significantly mediated by increased social integration and perceived social support from colleagues, friends, and family. These findings help to characterize the present psychological, emotional, and social wellness of classical musicians in the United States, the first step toward mitigating the hazardous impacts of COVID-19 on this vulnerable group's mental health and wellness.

## Introduction

On March 17, 2020, health authorities in the San Francisco Bay Area imposed the United States' first COVID-19 stay-at-home order (Allday, 2020). By the end of May 2020, 42 states and territories in the U.S. had enacted similar closures, dramatically disrupting the country's performing arts sector (A Timeline of COVID-19 Developments in 2020., 2021). As concert halls closed, performers faced heightened uncertainty about their careers. Particularly affected were classical musicians, who earn most of their income from live concerts and view performing as integral to their careers and identities (DiCola, 2013). The onset of COVID-19 closures required musicians to adapt creatively, moving performances from the concert hall to places ranging from porches, balconies, and the streets, to the internet (Gelt, 2021). As concert halls shuttered, musicians faced both professional and personal challenges—as was the case for much of the world, social distancing guidelines greatly isolated individuals, raising concerns for increased loneliness and mental health deficits. For musicians who usually performed with others in an ensemble, such solitude did not only result in loneliness—a key aspect of their music was inhibited.

Even prior to the pandemic, classical musicians faced intense competition and financial insecurity (Macnamara et al., 2014; Pecen et al., 2016; Ascenso et al., 2018), two intense occupational stressors that categorize the occupation as precarious work. Precarious work is defined as employment that is uncertain and unpredictable from the point of view of the worker (Kalleberg, 2009), causing far-reaching consequences to individuals' mental health and social outcomes (Kalleberg, 2011, 2018; Benach et al., 2014; Kelly et al., 2014; Schneider and Harknett, 2019). Indeed, such occupational challenges may contribute to a higher prevalence of mental health disorders in classical musicians than in the general population (Kegelaers et al., 2020). However, despite these occupational stressors, classical musicians tend nonetheless to maintain relatively high levels of satisfaction with their jobs and lives (Bonneville-Roussy et al., 2011; Brodsky, 2011). Many have even described performing as a lifestyle, rather than simply a means of earning a living (Oakland et al., 2012). This appears to hold true for the pandemic's disruptions as well; in recent interviews, classical musicians have described these disruptions to their career as bringing about “existential questions,” ranging from “how do we find meaning?” to “do we even continue to play music?” (Gelt, 2021).

The COVID-19 stay-at-home orders found many successful classical musicians unable to perform or earn a living from musical work, raising financial, social, and mental health concerns. A prior study of the pandemic and classical musicians analyzed the UK performing arts community, finding that classical orchestral musicians have been severely impacted by the closures enacted due to COVID-19 (Cohen and Ginsborg, 2021). The current study is, to our knowledge, the first analysis of how the COVID-19 pandemic has affected United States classical musicians' emotional experience, and how factors such as social relationships and loneliness mediate this experience. Given the intense social isolation brought by the pandemic, we frame our study around the important connections between social relationships and mental wellbeing (House et al., 1988; Turner and Marino, 1994; Thoits, 1995; Kawachi and Berkman, 2001; Schnittker, 2008; Wang et al., 2018), which are found to be important mediators to emotional and financial stress (Whelan, 1993; Wang et al., 2014).

From a sociological perspective, social relationships can be understood through three classes of phenomena: social integration, relational content, and social network structure (House et al., 1988). The present study focuses on the first two classes: social integration, defined as the quantity and type (e.g., kin/nonkin) of social ties, and relational content, defined as the functional quality of social relationships. An important aspect of relational content is social support, the positive, potentially stress-buffering aspects of relationships (Hall and Wellman, 1985). Given that the COVID-19 pandemic is a significant stressor to the global community (Bridgland et al., 2021; Whitehead, 2021), we position our study under Cohen and Wills' stress-buffering model of social support, wherein social support is hypothesized to prevent or modulate responses to stressful events that are damaging to health (Cohen and Wills, 1985). Under this model, social support may act on several points in the pathway between stressful events and harm to mental wellbeing, such as influencing an individuals' appraisal of the stressful situation or reducing a negative emotional reaction to stress (Cohen and Wills, 1985; Bailey et al., 1994; Kawachi and Berkman, 2001).

Given the existing literature, we hypothesized that increased measures of social integration and perceived social support would correlate with more positive measures of wellbeing in our study sample. We use validated measures of wellbeing to empirically analyze U.S. classical musicians' outlook on the future, emotional affect, and life satisfaction during the pandemic, focusing on the connections between social relationships and wellbeing.

## Materials and Methods

### Participants

Participants were recruited by email using the listservs of U.S. professional classical musician organizations such as small ensembles, orchestras, composers' associations, and chamber music groups. Inclusion criteria for the study were that participants must: (i) be a professional classical musician, defined as someone who makes the majority of their salary from classical music performances (U.S. Government., 1949), (ii) reside in the United States, and (iii) be over the age of 18. There was no specification of musical instrument detailed in participant criteria. Of the participants recruited for the study (*n* = 68), 32 identified as White, 10 identified as Black or African American, 16 identified as Asian or Asian American, 2 identified as Hispanic or Latinx, 2 identified as mixed-race or “other,” and 7 declined to report their race. Given previous data on the racial makeup of the classical music field, our data is mostly racially representative, though slightly overrepresents minority races (Doeser, 2016). 31 of the participants had completed a four-year college or conservatory program, 7 had started but not completed a four-year college or conservatory, 12 had completed a graduate or professional degree, 11 had graduated from high school or obtained a GED, and 8 declined to report their education. The median participant 2020 fiscal year total household income was $70,000. 52% of participants were married, 24% had living children, and 67% had other living immediate family. Upon survey completion, participants had the choice to enter their email address for an optional $10 Amazon gift card raffle.

### Measures

The survey was administered in March through May of 2021 using Qualtrics. This survey was comprised of validated measures of wellbeing designed to assess participants' time horizons, subjective wellbeing, social relationships, and a range of emotional experiences.

#### Time Horizons

Time horizons are defined as individual temporal strategies and orientations toward the past, present, and future (Lundqvist, 2020). We assessed time horizons with a modified version of the Future Time Perspective (FTP) scale (Carstensen and Lang, 1996). The original FTP scale consists of 10 statements about subjective time perception (e.g., “I could do anything I want in the future”), where participants rate how true each statement is for them on a 7-point scale from 1, *very untrue*, to 7, *very true*. This scale has been further adapted to occupational time horizons (Zacher and Frese, 2009; Henry et al., 2017), with prior findings suggesting that FTP at work mediates the relationship between occupational well-being and behavioral or motivational outcomes. In the present study, we adapted the occupational FTP model for classical musicians by altering the statements to pertain specifically to a musical career (e.g., changing “Many opportunities await me in the future” to “In my musical career, many opportunities await me in the future”). Additionally, we added three questions that concerned classical musicians' future planning, such as “I will challenge myself with new repertoire in the future.”

#### Subjective Wellbeing and Life Satisfaction

Subjective wellbeing refers to how individuals experience and evaluate their lives (Stone and Mackie, 2013). We assessed subjective wellbeing through the Diener Satisfaction with Life Scale (Diener et al., 1985, 2006, 2010). This scale assesses participants' satisfaction with their lives holistically, rather than with specific life domains (e.g., health or finances). It is a 5-statement survey, where participants indicate the degree to which they agree with each statement on a 7-point scale, with 1 being *strongly disagree* and 7 being *strongly agree*.

#### Social Integration and Relational Content

To assess participants' degree of social integration and perceived quality of relational content, we adapted previously validated questionnaires (Schuster et al., 1990; Turner and Marino, 1994), asking about both the quantity and quality of social ties through questions such as “How many musician colleagues do you regularly interact with professionally?” and “How close is your relationship with your musician colleagues?” For questions regarding the number of social or professional ties, several answer categories were presented, such as 0, 1–5, 6–10, 11–15, and 15+. For questions regarding the quality of social or professional ties, participants rated the closeness of their relationships on a four-point scale, where 1 indicated *very close* and 4 indicated *not at all close*. We further investigated the amount and degree of contact participants had with their social network, using the question “On average, how often do you communicate with musician friends and colleagues in the following ways?” with follow-up statements such as “rehearse or jam on-line” or “write or email.” For these questions about quantity of contact, participants answered each statement on a 6-point scale, with 1 indicating three *or more times a week* and 6 indicating *less than once a year or never*. Lastly, we further assessed participants' degree of social connectedness using the Social Support Convoy Model, which classifies participants' social connections as a network of social ties that provides protection and support (Kahn and Antonucci, 1980; Antonucci and Akiyama, 1987). This model asks participants to envision their social relationships as three separate levels of closeness and list the first names of people they believe fit into each level.

#### Positive and Negative Affect

Emotional experience was assessed with queries about the frequency of 29 emotions, 16 of which were positive and 13 of which were negative. We adapted this list of emotions from Carstensen et al. (2020), which measured the valence and arousal level of emotions of the general population during the COVID-19 pandemic. Participants were asked to rate how often they experienced each emotion during the past week on a 5-point scale, with 1 indicating *all or nearly all the time*, and 5 indicating *never*.

#### Effect on Employment

Participants were asked to indicate the extent to which their employment in a musical career had been impacted during the COVID-19 pandemic through one question, responding on a four-point scale, with 1 indicating *not at all* and 4 indicating *a great deal*.

### Ethical Considerations

All study procedures and analyses were approved by Stanford University's Institutional Review Board (IRB #59654), and informed consent was obtained from all participants. Other than the optional email address for the Amazon gift card, no identifying information was collected through the course of the study.

### Data Analysis

Statistical analyses were first conducted using R Core Team (2020). First, basic descriptive statistics (means, standard deviations, and frequencies) were calculated for all variables. After verifying assumptions of ordinarily and monotonicity (Wissler, 1905), Spearman's rank correlation coefficients (Savicky, 2014) were calculated to explore the direction and strength of potential relationships between emotional affect, FTP, life satisfaction, social relationships, and career effects. Then, after verifying that our observations were independent and have non-perfect separations, but are not normal, linear, or homoscedastic (Stoltzfus, 2011), we employed single and multivariate logistic regression analyses (R Stats Package, 2020; Wickham et al., 2021) to assess the relationship among loneliness, FTP, life satisfaction, and social relationships, controlling for potential demographic confounding variables such as race, socioeconomic status, and education level. Finally, to assess potential common variance bias, we used Harman's Single Factor Test in SPSS Version 28 (IBM Corp, 2021) (SPSS Statistics for MacOS, 2021).

## Results

### Emotional Affect

Participants' most commonly reported emotions were anxiety/worry, loneliness, and concern. These negative emotions were more common than any of the positive emotions. This is notable, given that surveys of the general population during COVID-19 find that participants report more positive than negative emotions (Carstensen et al., 2020). In our study, positive emotions were strongly positively correlated to other positive emotions, while negative emotions were weakly positively correlated to other negative emotions. Generally, positive emotions and negative emotions were negatively correlated. Of all 29 emotions assessed, the emotion of loneliness had the strongest negative correlation to positive emotions. Additionally, loneliness had the strongest negative correlation to measures of life satisfaction and shorter FTP.

### Social Integration and Relational Content

There is substantial evidence that low social integration and low degrees of perceived social are related to loneliness (Wang et al., 2018). To explore the emotion of loneliness further, we first examined the relationship between social integration as an independent variable and FTP, career, and life satisfaction as dependent variables using multivariate logistic regression. The question, “How many musician colleagues do you regularly interact with professionally?” was significantly related to all FTP questions, with the exception of the statement, “I could do anything I want in the future.” It was also significantly related to all life satisfaction questions, with increased musician colleague interaction associated with more positive life satisfaction. The number of musician colleagues was not associated with career satisfaction (Table 1). The question, “How many friends (musician or non-musician) do you have?” was significantly related to all FTP questions, with an increased quantity of friends correlating with a more positive FTP and increased life satisfaction. We were unable to interpret the relationship between “How many friends would you say you have a close relationship with?” and FTP or life satisfaction due to the ambiguity of participants' given answers (e.g., “many,” “few”). Quantity of friends was also significantly associated with career satisfaction (Table 2).

**Table 1:** Logistic regression between the question “how many musician colleagues do you regularly interact with professionally?” and measures of future time perspective (FTP), life satisfaction, and career satisfaction.

	**B**	***SE* B**	**β**	***T***	***p***
FTP 1	3.1317	0.3653	0.1673	3.159	0.00267^**^
FTP 2	3.4821	0.3732	0.1809	3.356	0.00150^**^
FTP 3	3.3856	0.4470	0.1099	2.510	0.0153^*^
FTP 4	3.1209	0.4351	0.0920	2.274	0.0272^*^
FTP 5	3.2221	0.4384	0.0650	1.884	0.0653
FTP 6	3.1789	0.4562	0.1096	2.505	0.0155^*^
FTP 7	5.7758	0.4438	0.2756	4.405	0.0000544^***^
FTP 8	5.1145	0.4734	0.1210	2.263	0.0115^*^
FTP 9	3.8798	0.3812	0.1949	3.153	0.000937^***^
Multivariate FTP	2.35377	1.15565	0.3895	2.037	0.007841^**^
Life satisfaction 1	3.5219	0.4382	0.0936	2.295	0.0259^*^
Life satisfaction 2	3.3916	0.4228	0.2151	3.738	0.000469^***^
Life satisfaction 3	3.3282	0.4861	0.1305	2.766	0.00788^**^
Life satisfaction 4	3.8366	0.3951	0.1677	3.174	0.00257^**^
Life satisfaction 5	3.6833	0.4516	0.1126	2.544	0.0140^*^
Multivariate life satisfaction	3.516	0.9338	0.2765	2.574	0.00895^**^
Career satisfaction	1.7718	0.42230	0.0467	1.581	0.120

*Both single variable and multivariate logistic regression analyses were conducted*.

* *indicates p < 0.05,*

** *indicates p < 0.01, and*

*** *indicates p < 0.001*.

**Table 2:** Logistic regression between the question “how many friends do you have?” and measures of future time perspective (FTP), life satisfaction, and career satisfaction.

	**B**	***SE* B**	**β**	***T***	***p***
FTP 1	3.42975	0.27022	0.1962	3.528	0.000897^***^
FTP 2	3.85573	0.28026	0.1884	3.441	0.00116^**^
FTP 3	3.23837	0.28556	0.3620	5.379	0.00000189^***^
FTP 4	3.09697	0.30023	0.2406	4.020	0.000192^***^
FTP 5	3.12205	0.30404	0.2099	3.681	0.000560^***^
FTP 6	3.17234	0.31070	0.2745	4.393	0.0000566^***^
FTP 7	5.0280	0.3505	0.2062	3.640	0.000636^***^
FTP 8	4.6992	0.3595	0.1099	2.484	0.0164^*^
FTP 9	4.08969	0.26424	0.3205	4.905	0.00000995^***^
Multivariate FTP	1.43030	0.95200	0.4327	3.559	0.00233^**^
Life satisfaction 1	3.60788	0.31311	0.1872	3.428	0.001212^**^
Life satisfaction 2	3.87624	0.31852	0.2174	3.764	0.000433^***^
Life satisfaction 3	3.42375	0.33586	0.2708	4.352	0.0000649^***^
Life satisfaction 4	4.25102	0.30054	0.1547	3.025	0.003924^**^
Life satisfaction 5	3.80499	0.32084	0.2133	11.869	0.0004933^***^
Multivariate life satisfaction	2.0147	0.9029	0.3144	4.219	0.00307^**^
Career satisfaction	1.58897	0.26836	0.4237	5.176	0.000008776^***^

* *indicates p < 0.05,*

** *indicates p < 0.01, and*

*** *indicates p < 0.001*.

We further analyzed the relational content of participants' social relationships through multivariate logistic regression using questions examining relationship quality and closeness as the independent variable and questions assessing FTP, life, and career satisfaction as dependent variables. The question, “How close is your relationship with your musician colleagues?” was significantly correlated with higher FTP scores but did not correlate with life satisfaction. However, the perceived quality of relationships with musician colleagues was significantly correlated with career satisfaction (Table 3). Answers to “How close is your relationship with your friends?” were significantly associated with FTP and life satisfaction, with closer relationships correlated with more positive scores on both. The quality of friendship was also correlated with career satisfaction (Table 4). Relationship quality with kin, as assessed with the question, “How close is your relationship with your family members?” was significantly correlated with all FTP questions, except for FTP8, which states “there are only limited possibilities in my future musical career.” Kin relationships were also correlated with career satisfaction (Table 5). However, in multivariate logistic regression analyses, family relationships were not correlated with life satisfaction.

**Table 3:** Logistic regression between the question “how close is your relationship with your musician colleagues?” and measures of future time perspective (FTP), life satisfaction, and career satisfaction.

	**B**	***SE* B**	**β**	***T***	***p***
FTP 1	2.5489	0.1855	0.2179	3.695	0.000555^***^
FTP 2	2.9046	0.1932	0.2192	3.709	0.000531^***^
FTP 3	2.6516	0.2343	0.1661	3.124	0.00230^**^
FTP 4	1.6855	0.2114	0.2961	4.540	0.0000367^***^
FTP 5	1.9450	0.2145	0.2406	3.941	0.000268^***^
FTP 6	2.1156	0.2307	0.2159	3.673	0.000592^***^
FTP 7	6.4058	0.6324	0.2554	4.099	0.000156^***^
FTP 8	6.0704	0.2485	0.1954	3.415	0.00131^**^
FTP 9	3.5934	0.2074	0.1572	3.023	0.000937^***^
Multivariate FTP	1.5474	0.8851	0.3514	1.322	0.0274^*^
Life satisfaction 1	3.3444	0.2432	0.1671	5.250	0.00297^**^
Life satisfaction 2	3.3581	0.2334	0.0125	2.652	0.111
Life satisfaction 3	3.1908	0.2713	0.0817	2.089	0.0673
Life satisfaction 4	3.3584	0.2123	0.5331	5.354	0.0107^*^
Life satisfaction 5	3.6386	0.2594	0.51626	3.053	0.0419^*^
Multivariate life satisfaction	1.1553	0.9419	0.1922	1.074	0.0841
Career satisfaction	1.4713	0.2331	0.05111	1.625	0.1107

* *indicates p < 0.05,*

** *indicates p < 0.01, and*

*** *indicates p < 0.001*.

**Table 4:** Logistic regression between the question “how close is your relationship with your friends?” and measures of future time perspective (FTP), life satisfaction, and career satisfaction.

	**B**	***SE* B**	**β**	***T***	***p***
FTP 1	2.1948	0.1800	0.2182	3.736	0.000481^***^
FTP 2	2.4357	0.1867	0.2363	3.933	0.000259^***^
FTP 3	2.2596	0.2259	0.1698	3.198	0.00240^**^
FTP 4	1.1991	0.2020	0.3047	4.680	0.0000222^***^
FTP 5	1.6684	0.2143	0.2065	3.607	0.000715^***^
FTP 6	1.4783	0.2209	0.2477	4.058	0.000174^***^
FTP 7	6.4987	0.7618	0.1866	3.387	0.00138^**^
FTP 8	6.2435	0.7462	0.1687	3.154	0.00275^**^
FTP 9	2.9316	0.1977	0.2108	3.655	0.000617^***^
Multivariate FTP	2.1948	0.1800	0.2182	3.736	0.00477^**^
Life satisfaction 1	1.5515	0.2012	0.3172	4.819	0.0166^*^
Life satisfaction 2	1.6491	0.1970	0.3697	5.416	0.0000138^***^
Life satisfaction 3	1.2020	0.2217	0.3409	5.085	0.00000176^***^
Life satisfaction 4	2.2851	0.1886	0.3090	4.681	0.00000554^***^
Life satisfaction 5	1.9825	0.2203	0.2535	4.121	0.0000229^***^
Multivariate life satisfaction	1.0762	0.3846	0.4286	2.798	0.0000903^***^
Career satisfaction	1.6917	0.2263	0.02006	1.012	0.020

* *indicates p < 0.05,*

** *indicates p < 0.01, and*

*** *indicates p < 0.001*.

**Table 5:** Logistic regression between the question “how close is your relationship with your family members?” and measures of future time perspective (FTP), life satisfaction, and career satisfaction.

	**B**	***SE* B**	**β**	***T***	***p***
FTP 1	2.5692	0.1741	0.2106	3.503	0.00104^**^
FTP 2	2.9735	0.1775	0.2066	3.461	0.00117^**^
FTP 3	2.0786	0.2026	0.2861	4.294	0.0000898^***^
FTP 4	2.2186	0.2100	0.1781	3.157	0.00281^**^
FTP 5	2.3725	0.2104	0.156	2.916	0.00547^**^
FTP 6	2.1680	0.2161	0.2082	3.478	0.00112^**^
FTP 7	5.5942	0.2481	0.1054	2.328	0.0243^*^
FTP 8	4.9835	0.2507	0.0473	1.495	0.142
FTP 9					0.00000827^***^
Multivariate FTP	0.2456	0.2308	0.4340	3.153	0.00639^**^
Life satisfaction 1	3.0951	0.2252	0.1077	2.356	0.00637^**^
Life satisfaction 2	3.4393	0.2250	0.1120	2.408	0.0228^*^
Life satisfaction 3	2.8638	0.2526	0.1224	2.533	0.0201^*^
Life satisfaction 4	3.7143	0.2060	0.1075	2.328	0.0148^*^
Life satisfaction 5	3.2371	0.2405	0.0987	2.244	0.0245^*^
Multivariate life satisfaction	1.4992	0.2505	0.1551	0.771	0.2093
Career satisfaction	0.8316	0.2117	0.1508	2.859	0.0297^*^

* *indicates p < 0.05,*

** *indicates p < 0.01, and*

*** *indicates p < 0.001*.

### Future Planning

We assessed the extent of participants' future planning with two questions: “I have an idea of what I will be doing musically 1 month from now” and “I have an idea of what I will be doing musically 6 months from now.” Most participants agreed with the first statement (M = 5.15, SD = 1.99, seven-point scale, with 7 indicating *very true* and 1 indicating *very untrue*) but disagreed with the second statement (M = 3.64, SD = 2.28, seven-point scale, with 7 indicating *very true* and 1 indicating *very untrue*). We further examined participants' degree of future planning with multivariate logistic regression, using 1-month and 6-months future planning as the independent variable and FTP, career, and life satisfaction as dependent variables. Both degrees of future planning were highly correlated with FTP scores. Additionally, future planning at both time points was highly correlated with degree of life satisfaction (Tables 6, 7). Interestingly, compared to 6-month future planning, 1-month future planning was less strongly correlated with the question, “compared to this time last year, I am more fulfilled by my career.”

**Table 6:** Logistic regression between responses to the statement “I have an idea of what I will be doing musically one month from now” and measures of future time perspective (FTP), life satisfaction, and career satisfaction.

	**B**	***SE* B**	**β**	***T***	***p***
FTP 1	2.7785	0.1004	0.1204	2.668	0.0102^*^
FTP 2	2.8475	0.0988	0.1823	3.405	0.00128^**^
FTP 3	2.2312	0.1112	0.2121	3.741	0.000458^***^
FTP 4	2.3765	0.1135	0.1302	2.790	0.00736^**^
FTP 5	2.0182	0.1060	0.1909	3.502	0.000956^***^
FTP 6	2.2215	0.1143	0.1717	3.283	0.00184^**^
FTP 7	6.3160	0.1221	0.1866	3.453	0.00111^**^
FTP 8	5.4046	0.1272	0.2082	2.135	0.0376^*^
FTP 9	2.8898	0.0955	0.2691	4.375	0.0000586^***^
Multivariate FTP	2.0867	0.3733	0.3283	2.523	0.00132^**^
Life satisfaction 1	2.3769	0.1078	0.2017	3.590	0.000742^***^
Life satisfaction 2	2.5955	0.1108	0.2148	3.736	0.000473^***^
Life satisfaction 3	1.8621	0.1169	0.2677	4.317	0.0000728^***^
Life satisfaction 4	2.9716	0.09916	0.2223	3.781	0.000418^***^
Life satisfaction 5	2.5763	0.1123	0.2018	3.591	0.000740^***^
Multivariate life satisfaction	5.0061	0.3934	0.4440	2.424	0.00203^**^
Career satisfaction	1.1301	0.1085	0.0835	2.156	0.0358^*^

* *indicates p < 0.05,*

** *indicates p < 0.01, and*

*** *indicates p < 0.001*.

**Table 7:** Logistic regression between the statement “I have an idea of what I will be doing musically six months from now” and measures of future time perspective (FTP), life satisfaction, and career satisfaction.

	**B**	***SE* B**	**β**	***T***	***p***
FTP 1	2.7089	0.3301	0.3408	5.185	0.00000360^***^
FTP 2	3.1857	0.0801	0.3046	4.773	0.0000152^***^
FTP 3	2.8809	0.0943	0.2659	4.340	0.0000659^***^
FTP 4	2.5410	0.0913	0.2705	4.391	0.0000556^***^
FTP 5	2.4970	0.0881	0.2757	4.460	0.0000441^***^
FTP 6	2.8018	0.0976	0.2170	3.796	0.000385^***^
FTP 7	5.4808	0.1075	0.1829	3.411	0.00126^**^
FTP 8	5.4583	0.1034	0.2273	3.874	0.000307^***^
FTP 9	3.5299	0.0795	0.3428	5.208	0.00000332^***^
Multivariate FTP	0.8988	0.2383	0.4534	3.964	0.000975^***^
Life satisfaction 1	2.5422	0.0802	0.4363	6.282	0.0000000738^***^
Life satisfaction 2	2.9299	0.0863	0.3931	5.747	0.000000510^***^
Life satisfaction 3	2.5858	0.0967	0.3592	5.347	0.00000212^***^
Life satisfaction 4	3.3659	0.0802	0.3624	5.331	0.00000235^***^
Life satisfaction 5	3.4088	0.1003	0.1870	3.426	0.00122^**^
Multivariate life satisfaction	5.0061	0.3934	0.4440	2.424	0.00000923^***^
Career satisfaction	1.0760	0.0878	0.2343	3.950	0.000240^***^

* indicates p < 0.05,

** *indicates p < 0.01, and*

*** *indicates p < 0.001*.

### Impacts on Employment

We assessed participants' employment during the pandemic using the question, “what describes your current employment status?” 87% of participants were currently working for pay (part time or full time), while the rest were currently unemployed. We also assessed the impact of COVID-19 on participants' employment with the question, “to what extent has your employment or retirement status been affected by the coronavirus pandemic?” using a five-point scale, with 1 indicating *not at all* and 5 indicating *a great deal*. The majority (67.3%) of participants answered *a great deal*. On average, participants' employment statuses were strongly affected by the coronavirus pandemic (M = 4.46, SD = 0.93). Surprisingly, neither 1-month nor 6-month future musical planning as an independent variable was correlated with employment status nor the impact of COVID-19 on employment status as dependent variables. Additionally, multivariate regression analysis of the impact of the pandemic on employment did not correlate to any measure of life satisfaction or FTP. However, it did have a significant relationship to career satisfaction (Table 8).

**Table 8:** Logistic regression between the question “to what extent has your employment or retirement status been affected by the coronavirus pandemic” and measures of future time perspective (FTP), life satisfaction, and career satisfaction.

	**B**	***SE* B**	**β**	***T***	***p***
FTP 1	3.7540	0.2199	0.0277	1.183	0.243
FTP 2	4.3071	0.2363	0.0051	0.500	0.619
FTP 3	3.8084	0.2654	0.0235	1.086	0.283
FTP 4	3.8959	0.2582	0.0014	0.260	0.796
FTP 5	3.5409	0.2536	0.0227	1.069	0.290
FTP 6	3.8462	0.2750	0.0071	0.591	0.557
FTP 7	4.5712	0.2941	0.0233	1.082	0.285
FTP 8	4.1760	0.2855	0.0032	0.395	0.695
FTP 9	4.4328	0.2362	0.0379	1.389	0.171
Multivariate FTP	1.7053	0.1482	0.1881	0.9485	0.434
Life satisfaction 1	4.2258	0.2581	0.0054	0.514	0.610
Life satisfaction 2	4.5053	0.2647	0.0094	0.681	0.499
Life satisfaction 3	3.9521	0.2994	0.0231	1.076	0.287
Life satisfaction 4	4.6561	0.2456	0.0071	0.584	0.562
Life satisfaction 5	4.8902	0.2777	0.0075	0.611	0.544
Multivariate life satisfaction	1.5297	0.1202	0.1324	1.440	0.264
Career satisfaction	1.1301	0.1085	0.0835	2.156	0.036^*^

* *indicates p < 0.05*.

## Discussion

This cross-sectional study revealed that the COVID-19 pandemic is negatively associated with the time horizons, life satisfaction, emotional experience, and overall wellbeing of professional classical musicians. The majority of musicians surveyed stated that the pandemic had affected their musical employment status a great deal, reporting that their careers during the pandemic were significantly less fulfilling than their careers prior to the pandemic.

Of the 29 emotions we assessed, the emotion negatively correlated to the greatest number of indicators for wellbeing was loneliness. Three general types of loneliness exist: situational loneliness, developmental loneliness, and internal loneliness. Situational loneliness refers to loneliness resulting from environmental factors and disasters, developmental loneliness results from personal inadequacies, developmental deficits, or poverty, and internal loneliness results from personality factors, mental distress, low self-esteem, and poor coping strategies with stress (Tiwari, 2013). While the loneliness experienced by participants may be classified under any one or multiple of these categories, the current COVID-19 pandemic is a significant environmental stressor that has affected every aspect of the world. Thus, it is not surprising that loneliness has been a defining characteristic of the COVID-19 pandemic, especially given the implementation of social distancing and “stay-at-home” orders for public health (Li and Wang, 2020; Luchetti et al., 2020).

Studies of the general population have found that not only has loneliness significantly surged during the pandemic (Killgore et al., 2020), it has also been associated with a breadth of mental health concerns, such as elevated rates of depression and higher suicidal ideation (Ingram et al., 2020). Even prior to the pandemic, loneliness has been linked to significant psychological health problems and found to increase the risk of distress, depression, anxiety, and suicidal ideation (Beutel et al., 2017). Beyond its mental health impacts, loneliness has also been known to have damaging effects on physical health, negatively affecting health behaviors, health care utilization, cardiovascular activation, cortisol levels, and sleep (Cacioppo et al., 2002). These physical health impacts can lead to disorders such as rheumatoid arthritis, lupus, cardiovascular disease, obesity, physiological aging, and cancer (Mushtaq et al., 2014). These negative mental and physical health effects of loneliness make our results especially concerning.

Though loneliness may be attributed to any number of causes, we posited that it may be related to the musicians' social relationships. Our findings supported this hypothesis, indicating that greater amounts of social integration and perceived quality of social support, whether it be from musician colleagues, friends, or family were negatively correlated with the emotion of loneliness. Both increased quantity and quality of social relationships appeared to yield this benefit, as well as correlate with higher scores on the FTP scale and greater life satisfaction. However, only perceived quality of kin relationships was correlated with career satisfaction, indicating that career and life satisfaction may be influenced by different variables.

Although the relationship between social support and mental wellbeing has been well-established in the sociological literature, we were surprised to find that while the quantity of musician colleague interaction was correlated with career satisfaction, the quality of these relationships was not. This may indicate that the mere presence of musical colleagues plays a vital role in the lives of musicians, even if these colleague relationships are not close. These results support prior work that found relational content to only partially mediate the impact of social integration on psychological wellbeing and mortality (Blazer, 1982). Additional research has also indicated that social integration is more consequential for health than is the perceived quality of relationships (House, 1984; House et al., 1988).

The next two most indicated surveyed emotions were anxiety/worry and concern. Anxiety during COVID-19 pandemic has been well characterized in multiple countries throughout the span of the pandemic. Even early on, social distancing greatly affected individuals' levels of anxiety: surveys of people quarantined in Wuhan, China during the first 2 weeks of lockdown (the first COVID-19 related lockdown in the world) found that the vast majority of respondents (70.78%) reported symptoms of anxiety (Cao et al., 2020). A similar phenomenon was seen in the U.S. at the onset of the pandemic, with anxiety levels significantly increasing compared to pre-pandemic times. However, in the general U.S. population, anxiety/worry has decreased in the most recent stages of the pandemic (spring and summer 2021), reverting to pre-pandemic levels (Li et al., 2021). Thus, it is interesting that the classical musicians surveyed in our study still report high levels of anxiety/worry and concern.

Our findings are in line with prior studies of classical musicians' anxiety, which have found that even pre-pandemic, anxiety is more prevalent in performing classical musicians than the general population (Barbar et al., 2014; Vaag et al., 2016; Kegelaers et al., 2020). This may be due to the precarious nature of the work, caused by occupation-specific stressors such as employment instability and performance anxiety (Ascenso et al., 2018). Our study finds that the COVID-19 pandemic has worsened employment stability for classical musicians, with the vast majority of respondents stating that their employment status has been affected *a great deal* by the pandemic. Job insecurity is linked to multiple aspects of mental health, including anxiety and worry (Menéndez-Espina et al., 2019). Thus, it is possible that pre-pandemic levels of anxiety/worry in classical musicians have been exacerbated by the addition of pandemic-specific occupational stressors.

Prior studies of COVID-19 and musicians have found that during the pandemic, classical orchestral musicians were overwhelmingly concerned about the future of their careers (Cohen and Ginsborg, 2021). Our investigation of musicians' time horizons through the FTP scale aligns with such findings. However, we also hypothesized that participants' time horizons may be moderated by their extent of future planning. Our results found that both 1-month and 6-month musical future planning were correlated with all measures of FTP and life satisfaction, indicating that both short- and long-term plans are related to musicians' wellbeing. Interestingly, only 6-month planning was correlated to career satisfaction, suggesting that long-term planning may have a unique relationship with a musical career. A potential explanation for this is the open-endedness of the question—we asked respondents their level of agreement with the questions, “I have an idea of what I will be doing musically one/six month(s) from now,” not limiting participants to musical plans within their employment. Thus, it is possible that participants referred to personal musical projects outside the scope of their primary employment. This may also explain the difference between 1-month and 6-month planning: it is more likely that musicians have scheduled performances 6 months in the future than 1-month in the future, given the present ongoing disruptions of the pandemic. Therefore, 6-month future planning may refer more directly to participants' primary careers, explaining the significant correlations between 6-month planning, positive FTP, and life satisfaction.

Interestingly, even though participants largely indicated that the pandemic had affected their employment status a great deal, most participants were actively employed at the time of survey. This indicates that participants' understanding of “affected employment status” may encompass more than layoffs. Throughout the pandemic, numerous U.S. orchestras and small ensembles furloughed their musicians. A prominent example is New York's Metropolitan Opera orchestra, whose members were furloughed without pay for months. Even after the furlough period ended, musicians were subjected to significant salary reductions (Jacobs, 2021). Numerous other ensemble groups around the country enacted similar pay-cuts to musicians, many of which are substantial and long-lasting, suggesting that employment disruptions will continue long after the pandemic has ended (Jacobs, 2020).

The results of our study find that the effects of the COVID-19 pandemic are significantly associated with classical musicians' views of their careers, time horizons, and wellbeing. At the time of writing, the pandemic continues to pose an ongoing threat to human health and society, even as vaccinations have become widely available in the United States. Fortunately, as social-distancing guidelines decrease, the performing arts sector has begun to return. However, the mere return of music to concert halls does not signify a solution to many classical musicians' challenges. The economic ramifications of the pandemic on the performing arts will be long-lasting, directly impacting musicians' livelihoods and careers. Thus, the challenges classical musicians are currently facing may outlast the pandemic, raising the question, “how do we best support this vulnerable group?”

One answer: help musicians cultivate resilience. Resilience is defined as the ability to withstand setbacks, adapt positively, and bounce back from adversity, all of which are vitally important in the face of increased stressors during the pandemic (Luthar and Cicchetti, 2000). Prior studies of classical musicians have found that increased psychological resilience is negatively correlated with mental health issues (Kegelaers et al., 2020), indicating its importance in our study population. Though studies of resilience in musicians are limited, prior findings suggest that resilience may be promoted by goal setting, increasing social connectedness, and creating a facilitative environment that reduces mental health stigma, increases mental health literacy, and encourages help-seeking behaviors (Polizzi et al., 2020; Wu et al., 2021). Our study finds that participants with increased goal setting (as measured through FTP), social integration, and perceived support demonstrated lower levels of negative emotions and higher levels of life satisfaction and wellbeing. Thus, it may be beneficial for classical musicians to employ goal-setting behaviors and increase social connectedness to increase psychological resilience. Likewise, ensemble groups and other musical organizations might consider implementing mental health resources and wellbeing workshops for their musicians.

### Study Strengths

The present study examines a vulnerable, yet greatly understudied population that is particularly positioned to be negatively affected by the COVID-19 pandemic. Using well-validated measures of wellbeing and social relationships, we draw important connections between social integration and support to time horizons, career satisfaction, and life satisfaction. Such findings reinforce prior sociological and psychological theory, emphasizing the great importance of social relationships in this unprecedented and uniquely stressful time.

### Limitations and Future Directions

Potential limitations of this study include our relatively small sample size, which reduces the external validity of the statistical findings. In general survey research, common method bias is a concern. However, we evaluated this possibility *post hoc*, employing Harman's single factor test using exploratory factor analysis. The total variance explained by a single factor was less than 41%, which falls below the threshold of 50%. Thus, while common method bias cannot be ruled out as a contributing factor in the present study, it does not appear to be a significant factor (Podsakoff et al., 2003). Since all data were self-reported, response bias may be a limitation to construct validity, given the potential influence of social desirability bias, recall bias, and demand characteristics. To reduce such bias, we ensured participants their responses would remain anonymous, asked only about events that took place within the last year, and did not reveal the goals nor hypotheses of the study during participant recruitment. In addition, though the COVID-19 pandemic presents a significant stressor to our study population, it is possible that participants may have had very different experiences during the pandemic. For instance, we did not ask musicians to specify their specific employer: it is possible that gig musicians had significantly less economic stability than ensemble musicians during the pandemic. Moreover, the cross-sectional design of our study precludes us from making a causal claim and reduces internal validity. Future studies could investigate the ramifications of COVID-19 on the classical musician population through increased sample size and longitudinal observations.

In conclusion, our study finds that the COVID-19 pandemic is associated with changes in nearly every aspect of U.S. classical musicians' lives, whether it be their careers, view of the future, emotional affect, life satisfaction, or overall wellbeing. Participants' most reported emotions were loneliness and anxiety, which have been defining emotional characteristics of the pandemic. Our results highlight the power of future planning and social connectedness to help benefit the emotional status, life satisfaction, and wellbeing of classical musicians, suggesting that psychological resilience may be an important and necessary protective factor against the stressors of COVID-19 and the classical music industry.

## Data Availability Statement

The raw data supporting the conclusions of this article will be made available by the authors, without undue reservation.

## Ethics Statement

The studies involving human participants were reviewed and approved by Stanford University Institutional Review Board (Protocol #59654). The patients/participants provided their written informed consent to participate in this study.

## Author Contributions

GW conceived the study and drafted the original manuscript, with revisions from JB, NF, and LC. All authors approved the submitted version.

## Funding

This work was funded by Stanford University School of Humanities and Sciences Faculty Research Awards.

## Conflict of Interest

The authors declare that the research was conducted in the absence of any commercial or financial relationships that could be construed as a potential conflict of interest.

## Publisher's Note

All claims expressed in this article are solely those of the authors and do not necessarily represent those of their affiliated organizations, or those of the publisher, the editors and the reviewers. Any product that may be evaluated in this article, or claim that may be made by its manufacturer, is not guaranteed or endorsed by the publisher.
