# Supplementary material for: Characterizing the Relationship Between the COVID-19 Pandemic and U.S. Classical Musicians' Wellbeing
Source: Front Sociol. 2022 Mar 24;7:848098. doi: 10.3389/fsoc.2022.848098 (PMC8988435; doi:10.3389/fsoc.2022.848098)
Supplement: Supplementary file 1 [file Data_Sheet_1.PDF]

SURVEY QUESTIONS GIVEN TO PARTICIPANTS
Survey given through Qualtrics in this order

Future time perspective

Lang, F. R., & Carstensen, L. L. (2002). Time counts: Future time perspective, goals, and social relationships. *Psychology and Aging, 17*(1), 125–139.
Henry H, Zacher H and Desmette D (2017) Future Time Perspective in the Work Context: A Systematic Review of Quantitative Studies. *Front. Psychol.* 8:413. doi: 10.3389/fpsyg.2017.00413

Read each item and, as honestly as you can, answer the questions: "How true is this of you?" All items pertain to your musical career. (on a scale of 1-7, where 1 means you believe that the statement is entirely false or highly unlikely, and 7 means you believe the statement is very true or very likely.)

1. In my musical career, many opportunities await me in the future.
2. I expect that I will set many new musical goals in the future.
3. Most of my musical career lies ahead of me.
4. There are infinite musical possibilities ahead in my future.
5. I could do anything I want in the future.
6. There is plenty of time left in my life to make new plans.
7. I have the sense time is running out.
8. There are only limited possibilities in my future musical career.
9. I will challenge myself with new repertoire in the future.
10. I have an idea of what I will be doing musically one month from now.
11. I have an idea of what I will be doing musically six months from now.
12. Compared to this time last year, I am more fulfilled by my career.

Life Satisfaction – Subjective Wellbeing

Diener, E., Emmons, R. A., Larsen, R. J., & Griffin, S. (1985). The Satisfaction with Life Scale. Journal of Personality Assessment, 49(1), 71-75. Pavot, W., & Diener, E. (1993). Review of the Satisfaction with Life Scale. Psychological Assessment, 5(2), 164-172.
Diener, E., Lucas, R. E., & Scollon, C. (2006). Beyond the hedonic treadmill: Revising the adaptation theory of well-being. American Psychologist, 61(4), 305-314. Diener, E., & Seligman, M. E. P. (2004). Beyond money: Toward an economy of well-being. Psychological Science in the Public Interest, 5(1), 1-31. 19 Diener, E., Suh, E. M., Lucas, R. E., & Smith, H. L. (1999). Subjective well-being: Three decades of progress. Psychological Bulletin, 125(2), 276-302. Stone, A.A., Schwartz, J.E., Broderick, J.E., Deaton, A. (2010). A snapshot of the age distribution of psychological well-being in the United States. Proceedings of the National Academy of Sciences of the United States of America, 107(22), 9985-9990.

Please say how much you agree or disagree with the following statements.
(7 point scale: 1 = Strongly disagree, 2 = Somewhat disagree, 3 = Slightly disagree, 4 = Neither agree nor disagree, 5 = Slightly agree, 6 = Somewhat agree, 7 = Strongly agree)

13. In most ways my life is close to ideal.
14. The conditions of my life are excellent.
15. I am satisfied with my life.
16. So far, I have gotten the important things I want in life.
17. If I could live my life again, I would change almost nothing.

Social Network, relationship quality, social support

Schuster, T. L., Kessler, R. C., & Aseltine, R. H. Jr. (1990). Supportive interactions, negative interactions, and depressed mood. American Journal of Community Psychology, 18, 423-438. Turner, R. J., Frankel, G., & Levin, D. M. (1983). Social support: Conceptualization, measurement, and implications for mental health. In J. R. Greenley & R. G. Simmons (Eds.), Research in Community and Mental Health (pp. 67-111). Greenwich: JAI Press.
(1-4 Scale for Q22, 23, 25-27, 1 = Very close, 2 = Quite close, 3 = Not very close, 4 = Not at all close)

18. Do you have a husband, wife, or partner with whom you live?
19. Do you have any living children?
20. Do you have any other immediate family, for example, any brothers or sisters, parents, cousins or grandchildren?
21. How many musician colleagues do you regularly interact with professionally? (0, 1-5, 6-10, 11-15, 15+)
22. How many non-musician colleagues do you regularly interact with professionally? ** (0, 1-5, 6-10, 11-15, 15+)
23. How many friends do you have?
24. How close is your relationship with your spouse or partner?
25. How close is your relationship with your family members?
26. How many friends would you say you have a close relationship with?
27. How close is your relationship with your friends?
28. How close is your relationship with your musician colleagues?
29. How close is your relationship with your non-musician colleagues?

On average, how often do you communicate with musician friends and colleagues in the following ways? (Contact with social network)
(1-6 Scale, 1 = Three or more times a week, 2 = Once or twice a week, 3 = Once or twice a month, 4 = Every few months, 5 = Once or twice a year, 6 = Less than once a year or never)

30. Rehearse or jam on-line
31. Rehearse or jam live but socially-distanced
32. Meet up (include both arranged and chance meetings)
33. Speak on the phone
34. Text message or another form of instant messaging
35. Write or email
36. Communicate via Zoom, FaceTime, Skype, or another video conferencing platform
37. Communicate via Facebook, Instagram, Twitter, or another social media platform

Social Support Convoy Model

Think of the three circles below as including people who are important in your life right now, but who are not equally close. Read the descriptions below and list the **first names** of the people you think fit into each circle based on how close you feel with that person.

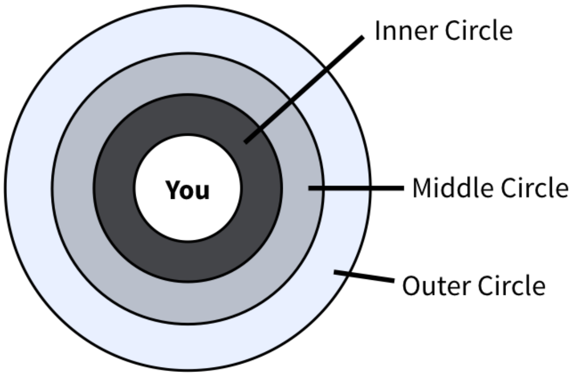

Inner Circle: *People to whom you feel so close that it is hard to imagine life without them.*

Middle Circle: *People to whom you may not feel quite that close but who are still important to you.*

Outer Circle: *People whom you haven't already mentioned but who are close enough and important enough in your life that they should be placed in your personal network.*

Antonucci, T. C. (1986). Measuring social support networks: Hierarchical mapping technique. Generations, 10-12.

Positive and Negative Affect

Watson, D., & Clark, L.A. (1994). The PANAS-X: Manual for the positive and negative affect schedule – expanded form. University of Iowa. http://ir.uiowa.edu/psychology_pubs/11/
https://www2.psychology.uiowa.edu/faculty/clark/panas-x.pdf

Carstensen, L. L., Pasupathi, M., Mayr, U., & Nesselroade, J. R. (2000). Emotional experience in everyday life across the adult life span. Journal of Personality and Social Psychology, 79(4), 644-655. Ong, A.D., Edwards, L.M., & Bergeman, C.S. (2006). Hope as a source of resilience in later adulthood. Personality and Individual Differences. 41(7), 1263-1273.

(1-5 Scale, 1 = All or nearly all of the time, 2 = Most of the time, 3 = Some of the time, 4 = Rarely, 5 = Never)

38. During the past week, to what degree did you feel:
a) Affectionate?
b) Energetic?
c) Accomplished?
d) Angry?
e) Interested?
f) Calm?
g) Appreciative?
h) Content?
i) Disgusted?
j) Quiet?
k) Sad?
l) Embarrassed?
m) Bored?
n) Anxious/worried?
o) Relaxed?
p) Fearful?
q) Peaceful?
r) Ashamed?
s) Relieved?
t) Guilty?
u) Happy?
v) Proud?
w) Amused?
x) Joyful?
y) Irritated?
z) Frustrated?
aa) Excited?
bb) Concerned?
cc) Lonely?

Adapted from Smith, J., Fisher, G., Ryan, L., Clarke, P., House, J., & Weir, D. R. (2013).Psychosocial and Lifestyle Questionnaire 2006–2010: Documentation re-port: Core section LB[Documentation of psychosocial measures in theHealth and Retirement Study]. Ann Arbor, MI: Survey Research Center,Institute for Social Research, University of Michigan.

39. What best describes your race/ethnicity? *(Check all that apply)*
   - o Asian/Asian-American
   - o Black/African-American
   - o Hispanic/Latin American/Latinx

- Middle eastern or North African
- Native American/Alaska Native
- Native Hawaiian or Other Pacific Islander
- White/European-American
- Another race or ethnicity not mentioned here (please describe, open-ended)
- Prefer not to say

40. What is the highest level of education you have completed?
    - Less than high school
    - Graduated high school (or GED)
    - Some college, conservatory, or technical school
    - Completed 4-year college/conservatory (BA, BS, BFA)
    - Completed graduate or professional degree
41. Which of the following best describes your total household (family) income from all sources <u>last year, before taxes?</u>
    - Less than $10,000
    - $10,000 to $20,000
    - $20,000 to $30,000
    - $30,000 to $40,000
    - $40,000 to $50,000
    - $50,000 to $60,000
    - $60,000 to $80,000
    - $80,000 to $100,000
    - $100,000 to $120,000
    - $120,000 to $140,000
    - $140,000 to $160,000
    - $160,000 to $180,000
    - $180,000 to $200,000
    - $200,000 to $220,000
    - $220,000 to $250,000
    - Greater than $250,000
    - Decline to answer
42. What best describes your current employment status?
    - Working for pay (part-time or full-time)
    - Not currently working for pay
    - Retired
43. To what extent has your employment or retirement status been affected by the coronavirus pandemic/
    - Not at all
    - A little
    - Somewhat
    - A moderate amount
    - A great deal
